# Enhancement of Speed and Accuracy Trade-Off for Sports Ball Detection in Videos—Finding Fast Moving, Small Objects in Real Time

**DOI:** 10.3390/s21093214

**Published:** 2021-05-06

**Authors:** Alexander Hiemann, Thomas Kautz, Tino Zottmann, Mario Hlawitschka

**Affiliations:** 1 Institute of Computer Science, Leipzig University, 04109 Leipzig, Germany; 2 Machine Learning and Data Analytics Lab, Department Artificial Intelligence in Biomedical Engineering (AIBE), Friedrich-Alexander-Universität Erlangen-Nürnberg (FAU), 91052 Erlangen, Germany; thomas.kautz@fau.de; 3 Media Seasons, 04105 Leipzig, Germany; Zottmann@MediaSeasons.com; 4 Faculty of Computer Science and Media, Leipzig University of Applied Sciences, 04277 Leipzig, Germany; mario.hlawitschka@htwk-leipzig.de

**Keywords:** sports ball detection, small object detection, YOLO, data augmentation, speed-accuracy trade-off

## Abstract

The detection and localization of the ball in sport videos is crucial to better understand events and actions occurring in those sports. Despite recent advances in the field of object detection, the automatic detection of balls remains a challenging task due to the unsteady nature of balls in images. In this paper, we address the detection of small, fast-moving balls in sport video data and introduce a real-time ball detection approach based on the YOLOv3 object detection model. We apply specific adjustments to the network architecture and training process in order to enhance the detection accuracy and speed: We facilitate an efficient integration of motion information, avoiding a complex modification of the network architecture. Furthermore, we present a customized detection approach that is designed to primarily focus on the detection of small objects. We integrate domain-specific knowledge to adapt image pre-processing and a data augmentation strategy that takes advantage of the special features of balls in images in order to improve the generalization ability of the detection network. We demonstrate that the general trade-off between detection speed and accuracy of the YOLOv3 model can be enhanced in consideration of domain-specific prior knowledge.

## 1. Introduction

Data analysis in sports has garnered significant attention in recent years. A growing awareness of the capabilities of data analysis and statistics as well as the availability of new tracking technologies are driving forces for this evolution. The analysis of position and motion data in sports does not only provide important insights for training purposes but is also an increasingly important source of information for spectators’ engagement and broadcast enhancements at professional sports events [1]. Especially during times of COVID-19 and the consequent exclusion of spectators, sports events lose their most important emotional component. Thus, new data analysis and broadcast enhancement technologies can enrich the viewing experience and help to increase the attraction of sport.

Balls are the major point of attention in many sports and games. In these ball sports, the position of the ball is a valuable input for tactical considerations, training methodology studies or other analyses and evaluations that aim for a better understanding of events and actions occurring in those sports. A common tool for the determination of the ball’s position is tracking of ball movements in ball based sports video sequences or in video streams gathered from cameras that capture the field of play. Detection of the ball in images is the first and potentially the most important step of ball tracking [2].

Despite recent advances in the field of computer vision, the detection of fast moving, small objects like balls in images remains a challenging task [3]. Compared to the detection of larger objects in images, the performance of small object detection is still relatively low [4]. The detection of balls in particular is subject to manifold difficulties: Considering sports video footage, sport balls often appear as small, fast moving objects that need to be detected in large and complex scenes. Continuously changing backgrounds as well as motion blurring and frequent changes in size, position, and orientation impair the detection and, accordingly, the tracking processes [5]. Balls cover only a small area of the entire input image. Accordingly, the training of object detectors for ball detection is a complex task, as the small ground truth objects overlap the predicted bounding boxes of the detector only occasionally. In contrast to general object detection tasks where just a few images contain small objects, balls are present in a large number of captured images, since the ball is often the object of interest in the respective sports. For most sports, merely one ball is present in an image, which often covers only specific areas in a captured scene. Accordingly, a ball detection model needs to be trained on a great number of images that capture the ball under different conditions and in various backgrounds before it is able to generalize.

In addition to the accuracy of detection, the detection speed plays a critical role in various application areas. In order to achieve a real added value for the use of ball tracking systems for fan engagement or the application in training systems, ball position information must be available immediately, without noticeable delays. Therefore, the respective detection algorithms must work in real time, optimally with processing speeds of more than 30 frames per second [6]. For many modern state-of-the-art object detection systems, the adaption to a specific detection task involves a trade-off between detection speed and accuracy. Since ball detection requires both high accuracy and high speed, the mastery of this trade-off represents an extremely complex task.

A specificity of ball detection is that the sports balls often appear as rather abstract objects in the respective video footage, which have hardly any special visual characteristics. This is partly caused by the low resolution of these balls in the image data. Details on the surface of a ball disappear due to undersampling in image space. Furthermore, fast moving balls are often prone to motion blurring due to high exposure times. In some sports, for example with a single-colored ball, the ball itself has no special visual texture. In these cases, the detection task is limited to the recognition and localization of monochromatic, circular blobs in the image.

Due to these special characteristics, the ball is not even clearly visible to the human eye, when individual images are considered. In contrast, when video sequences are examined, it is easier for the human observer to identify the ball in a single frame. Many of the widely used detection models perform the detection task on individual images. They treat video object detection as separated detection steps on individual images in a sequence of images. It is often ignored that these images cannot be viewed independently, since they are temporally related.

In this paper, we present an enhancement of the speed-accuracy trade-off for the detection of small, fast-moving objects by enabling accurate detection while preserving fast processing speed:

We introduce a real-time ball detection approach based on the YOLOv3 object detection model and apply specific adjustments for the detection of balls: First, we present an adapted multi-resolution feature extraction which is designed to find primarily small objects in the image. In order to improve detection accuracy, we integrate domain specific knowledge to improve image pre-processing. Second, we add the temporal dimension to the problem of ball detection by facilitating an efficient integration of motion information, while avoiding complex modifications of the network architecture. Third, we implement a data augmentation strategy that takes advantage of the special features of balls in images in order to enhance the generalizability of the detection network. Finally, we optimize the detection models for high-performance inference in order to use them for real-time applications.

## 2. Related Work

### 2.1. Object Detection in Images

Object detection refers to a classical task in computer vision which comprises the integrated localization and classification of objects in images [7]. The localization of the detected objects can be achieved in different ways. Simpler approaches are limited to the detection of bounding boxes around the detected objects or the abstraction of the object positions to a single point. Segmentation methods, on the other hand, provide image masks containing all pixels that belong to a specific object.

Object detection, as one of the central problems in computer vision, has been an active field of research for many years. Traditional object detection models typically apply a pipeline of three different stages: region selection, feature extraction, and classification. Region selection algorithms are applied to extract possible positions of objects in the image. The generation of candidate regions is mainly based on sliding window methods or subsets of regions in the image. Often times, this is inefficient and inaccurate or produces redundant candidate regions [7]. Feature extraction methods, such as SIFT [8], HOG [9], or Haar-like methods [10], are applied to extract robust visual features that describe the objects in the image. Finally, classificators, such as SVM [11] or Viola-Jones [12], are used to identify objects in the image and to distinguish different object classes. However, the combination of manually designed feature descriptors and shallow classification models is often not sufficient to solve complex detection tasks [7].

Due to the increase in computing power of the required training hardware and especially the availability of large annotated data collections [13,14], deep learning-based detection models have evolved to state-of-the-art approaches for object detection during the last decade. These detection models are typically based on convolutional neural networks (CNN) and have deeper architectures and, therefore, a higher capacity to learn complex features than traditional models. The application of deep learning algorithms allows for learning meaningful object representations without having to design features manually.

Among the variety of specific models for object detection, some fundamental detection model architectures have become generally accepted. State-of-the-art object detector architectures can mainly be divided into two groups: two-stage and single-stage detectors [15]. Two-stage detectors, such as Faster-RCNN [16] and Mask-RCNN [17] or R-FCN [18], treat object detection as a two-step process. At a first stage, a Region Proposal Network proposes a set of bounding boxes which may probably contain relevant objects. In a second stage, these proposals are classified using a common deep learning-based classification architecture.

Single-stage or single-shot detectors, such as SSD [19] or models from the YOLO model family [6,20,21], combine the detection and classification step in a single stage by treating the bounding box and class probability predictions as a regression task. Instead of an additional network that generates region proposals, single stage detectors use predefined anchors which represent different spatial positions, sizes, and aspect ratios of potential objects distributed over the input image. Object bounding box coordinates and class probabilities are predicted relative to these predefined anchors.

The selection of an appropriate architecture for a detector to handle a specific detection problem is not a straightforward task. It is always a trade-off between accuracy and speed [22]. Two-stage detectors are typically characterized by high accuracy rates, but at the cost of decreased detection speed. In contrast, single-stage models achieve lower accuracies but work with significantly higher speeds since all computations are integrated in a single network. Therefore, single-stage object detectors are even able to achieve real-time capability under certain circumstances.

Common object detection models are designed to provide valuable information for the semantic understanding of an image. Thus, these models are trained to recognize different types of objects and to locate them within an image. For more specific detection tasks, such as ball detection, where the object of interest is defined in advance, it is not required to detect a multitude of different object classes. Instead, these predefined object types should be detected reliably under changing conditions. Existing works in the field of ball detection [23,24] re-train existing detection model architectures on custom data sets. However, they limit the adaption of the detection networks, such as YOLO [20], to the simple adjustment of hyperparameters, e.g., the adaption of the network input resolution. Due to prior knowledge about the characteristics of balls in image data, there is room to enhance detection, both in terms of accuracy and speed, by adapting the network architecture and the training process for ball detection.

A large number of papers focus exclusively on the improvement of the detection accuracy. Only a small portion of previous research in the field of object detection focuses on the processing speed as a feature of detection, for example R-FCN [18], SSD [19], and YOLO [21]. However, this consideration is often limited to a simple statement about the latency or speed of detection. Thus, several papers deal with the development of guidelines for the appropriate selection of a detection network architecture that achieves the right balance between speed and accuracy [15,22] for a given detection task. Thereby, the trade-off between speed and accuracy depends primarily on the choice of the network meta-architecture, the selected feature extractors, and other relevant parameters, such as the input image size [22]. Only limited attention has been paid to the enhancement of this trade-off with regard to the specific task of ball detection to enable both fast processing speeds and accurate detection.

One approach to overcome the problems of small object detection is the augmentation of training image data by oversampling images with small objects. Each image gets augmented by copy-pasting small objects many times [25] in order to increase the occurrence and the diversity of locations of these objects in the training images. Another common approach are methods for synthesizing images to increase the size of the training dataset [26]. We adapt these approaches in order improve the training of the ball detection model.

### 2.2. YOLOv3

**Figure 1:**
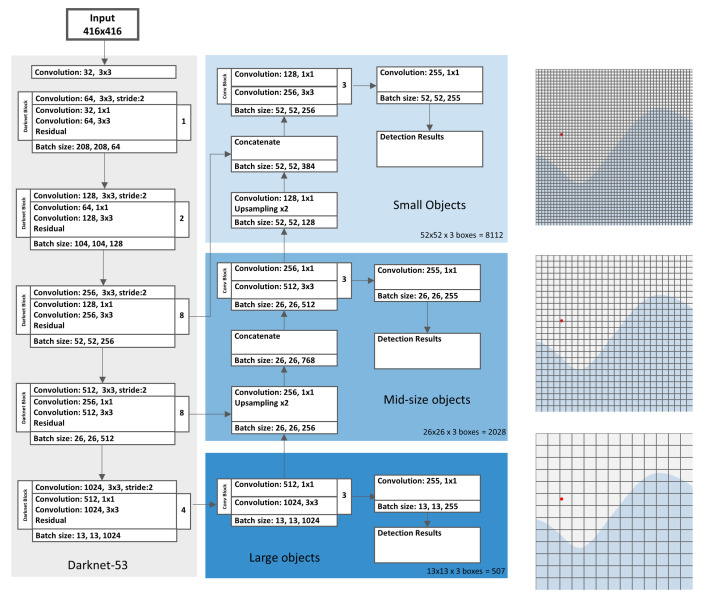
Architecture of the original YOLOv3 with 416 × 416 pixels input resolution.

In this paper, we use YOLOv3 (Figure 1) as the baseline for adaptions to the specific task of ball detection. In order to be able to better describe the subsequent enhancement steps, we briefly introduce the general concept of YOLOv3 and explain the features that are important for the subsequent modifications.

The YOLO model family is a popular family of object recognition models for detection in real-time. Generally, YOLO uses a single convolutional neural network to predict bounding boxes and class probabilities from an image in a single regression step. The network can be trained end-to-end to predict box coordinates and class label for each bounding box directly.

YOLOv3 is an “incremental improvement” on YOLOv2 [20], introducing a set of design changes that were inspired by recent advances in the object detection world [27]. YOLOv3 retains the essential characteristics of YOLOv2, but introduces updates that significantly enhance the performance of the detection of small objects.

Darknet-53: YOLOv3 uses a neural network to perform feature extraction, called Darknet-53. Darknet-53 has 53 convolutional layers instead of 19 in its previous version. Darknet-53 is based on successive 3 × 3 convolutions for feature extraction and 1 × 1 convolutions for the reduction of the output channels. Furthermore, shortcut connections are added, comparable to the residual network in ResNet [28].

Detection across 3 scales: One architectural change that significantly enhances the detection performance on small objects is the introduction of multi-scale predictions. Similar to feature pyramid networks (FPN) [29], YOLOv3 predicts bounding boxes at three different scales. To achieve this, several convolutional layers are added to the Darknet-53 feature extractor network forming three branches, each responsible for the detection of objects at a different scale. The scales are defined by downsampling the dimensions of the input image by 32, 16, and 8, respectively. Each branch uses information from different parts of the feature extractor network. The last branch uses information from the last layers of Darknet-53 where the input data are downsampled by the factor 32. The respective feature map is used to predict the boxes for larger objects in the input image. The two other branches further use information from previous layers in Darknet-53 in order to preserve finer-grained information. Feature maps from the rear layers of the network are upsampled and concatenated with previous feature maps in the network. This method allows for using meaningful semantic information from upsampled features and to preserve finer-grained information from earlier feature maps [21]. The combination with finer-grained information facilitates better detection of small objects.

YOLOv3 only uses convolutional layers for prediction, making it a fully convolutional network (FCN). No pooling layers are used. Instead, convolutional layers with stride 2 are applied to downsample the feature maps. This prevents the loss of low-level features that are required for the detection of small objects. Furthermore, it allows for changing the input image resolution without any changes on the network architecture. Accordingly, YOLOv3 is invariant to the size of the input image. For example, if height and width of the input image are doubled, the number of output grid cells gets quadrupled and, accordingly, four times more boxes are predicted.

As the network downsamples the input data by a factor of 32 at maximum (in the last branch), the given input image dimensions should be a multiple of 32. With the standard input resolution of 416 × 416 pixels, the produced output feature maps have dimensions of 52 × 52, 26 × 26, and 13 × 13 pixels for the respective scales.

The output of YOLOv3 is generated by applying 1 × 1 detection kernels on feature maps at the last convolutional layers in the respective scale branches. The predicted 3D tensor encodes the respective offsets for the predicted bounding boxes, the objectness scores, and the class predictions [20]. For YOLOv3, trained on the MS COCO dataset, the tensor encodes the class probabilities for all 80 classes in the dataset. At each scale, three boxes are predicted for every grid cell. Accordingly, the tensor dimensions are given by N×N×[3×(4+1+80)] at each branch, where N is the feature map size at the respective scale. For each detection box, four bounding box offsets, the objectness score, and 80 class probabilities are calculated. The total number of predicted boxes for YOLOv3 trained on the COCO are 10,647: 13×13×3=507 boxes in the big objects branch, 26×26×3=2028 boxes in the mid-size objects branch, and 52×52×3=8112 boxes in the small objects branch (see Figure 1).

Non-Maximum Suppression: YOLO applies thresholding to the objectness scores of the predicted bounding boxes in order to filter out boxes having scores below a certain objectness score. Generally, this threshold is set to 0.5. However, it is possible that the YOLO detection network detects more than one bounding box for the same object. In order to avoid duplicated detections in the final predictions, YOLO applies non-maximum suppression (NMS) to remove duplications that have lower confidences.

Input image size: Merely convolutional layers are used for prediction, which allows for changing the input image resolution without any changes on the network architecture. Accordingly, YOLOv3 is invariant to the size of the input image. The variable input size allows for an easier trade-off between speed and accuracy. Accordingly, a higher input resolution may potentially facilitate the detection of smaller objects, taking into account a possible loss of detection speed.

Anchor Boxes: Analogous to Faster R-CNN, YOLOv3 uses the concept of anchor boxes (or prior boxes). Anchor boxes are pre-defined bounding boxes that represent the shapes and sizes of objects that are present in the training data set. The detection network uses these pre-defined boxes in order to predict offsets on how to move and reshape the anchor boxes relative to a grid cell instead of predicting the actual position and size of a bounding box directly. In that sense, anchor boxes are a tool to tune the network to detect objects of a given size and shape. If the predefined anchor boxes are not selected correctly for the given data, the network may not know about the existence of specific irregular sized objects and is therefore not able to detect these objects correctly. At each scale, YOLOv3 uses three anchor boxes for predictions, accordingly nine boxes in total.

YOLOv3 is able to learn how to adjust the anchor boxes appropriately independent of the specified sizes and shape. However, it is easier for the network to learn if the specified anchor boxes represent the data set well. Instead of a manual selection of prior boxes, the selection of appropriate anchor boxes for YOLOv3 is done prior to the training process by analyzing the training dataset using k-means clustering.

### 2.3. Object Detection in Videos

Although established detection methods have largely focused on the processing of individual images, the publication of research papers dealing with detection in video data has increased in recent years. Especially since the release of large-scale video datasets, such as ImageNet VID [30], more research focused on the integration of the temporal component.

One way to integrate additional temporal information without changing the actual architecture of the detection network is to modify the post-processing step of the object detection pipeline. Methods, such as Seq-NMS [31], link the detection results of successive images and modify the detection confidences based on previous results in order to avoid mis-detections and to stabilize the detection quality. The actual detection is still performed per frame, but these methods require overlapping of object bounding boxes in successive frames. Since balls can change their position quickly and unpredictably within two consecutive frames, these methods are unsuitable for the application in robust ball tracking applications.

As the integration of temporal information adds another dimension to the detection problem, the application of 3D convolutions is an intuitive solution. 3D convolutional approaches [32,33,34]) have proven their ability to extract features for spatio-temporal data, especially for action recognition and image segmentation, and can be transferred to video object detection tasks. Even though the accuracy and stability of detection might get improved by the application of 3D convolutions, the complexity of the underlying models and the high computational effort for the detection using multi-dimensional matrices does not allow processing speeds of 30 FPS or more and inhibits real-time processing.

A common solution to consider the temporal context of a data series is the application of recurrent neural networks, such as LSTMs and GRUs. Recent video detection approaches that incorporate recurrent neural networks proved increased detection accuracy compared to static methods [35] and can even run at higher frame rates [36]. However, these methods are commonly trained end-to-end and require a huge amount of related annotations of continuous video sequences for the model training. Accordingly, existing large datasets for single image object detection can only be used for transfer learning to a limited extent. To train on custom video data, the data annotation process is more complex and time-consuming compared to static image object detection methods.

The most explored field that takes advantage of the temporal dimension of video object detection is optical flow. Optical flow estimates the visible motion of objects in two consecutive frames in a video. The outputs of dense optical flow methods are usually two-dimensional vector fields where each vector represents the displacement of a pixel from one image to its successor. In recent years, traditional methods, such as the Lucas–Kannade method [37], have been replaced by deep learning-based approaches, such as variants of FlowNet [38,39], SPyNet [40], or PWC-Net [41]. Approaches that compute the optical flow using deep neural networks generally take consecutive frames as the input and output the optical flow as color coded images representing the optical flow field.

Scientific works, such as that of Zhu et al. [42,43,44], utilize optical flow computation to create feature connections across multiple frames in order to improve accuracy or speed of object detection in videos. They integrate optical flow to increase the inference speed for video object detection. Most state-of-the-art object detectors are too slow to be applied to every frame of an input video. Zhu et al. [42] use the concept of sparse key frames. The input video is divided into image sequences with fixed lengths. The expensive object detector is exclusively applied on the first frame in each sequence, called the key frame. The deep feature maps of the detector are propagated to the subsequent frames via a flow field. Since flow computation methods are relatively fast compared to common object detectors, this approach achieves a significant speedup for inference.

Zhu et al. [43] use flow-guided feature aggregation across multiple frames to increase the accuracy of video object detection. A feature extraction network is applied on individual frames to predict the individual feature maps. An optical flow network estimates the motion between nearby frames. The flow information is used to warp feature maps from nearby frames and aggregate them relative to a given reference frame. These aggregated feature maps are passed to a detector, which calculates the detection results for the respective reference frame.

Even though these methods are even trainable end-to-end, the computation of optical flow using deep neural networks requires large amounts of training data. Furthermore, these methods are often designed for large and mid-sized objects in videos that do not show larger displacements and significant changes in appearance between subsequent frames. For small and fast moving objects, however, these methods achieve relatively poor results [43].

## 3. Methods

### 3.1. Adaptions of YOLOv3

**Figure 2:**
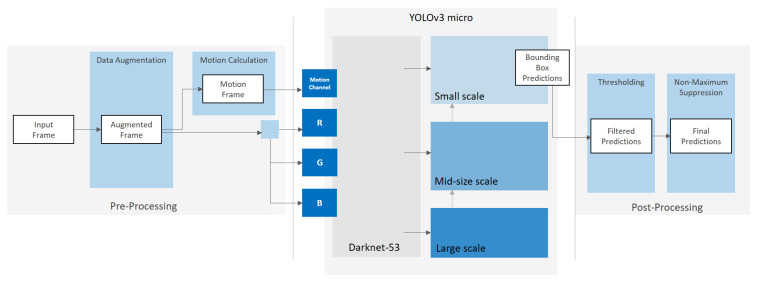
High-level overview of the adapted YOLOv3 training and inference pipeline. For training, we apply data augmentation to every frame by inserting additional balls. For each frame, we consider the previous images to calculate motion information. The value channel of the resulting motion frame is added to the network in addition to the original RGB image. We limit the bounding box prediction to the small objects branch in order to focus detection on small objects only.

YOLOv3 is a general purpose detector that is designed to detect different object types simultaneously [6]. However, for specific, single class detection tasks, such as the detection of balls, the detector can be highly customized using prior knowledge. The more restrictions and adjustments can be implemented for a specific detection task, the less the detector has to deal with variations. Even though YOLOv3 is better at detecting small objects than its previous versions, there is still room to improve the detection on small, fast moving objects. In the following paragraphs, we introduce different adjustments to the standard YOLOv3 architecture and training process (Figure 2) that are designed to enhance the detection accuracy, while preserving sufficient processing speed for real-time applications.

### 3.2. Input Size

**Figure 3:**
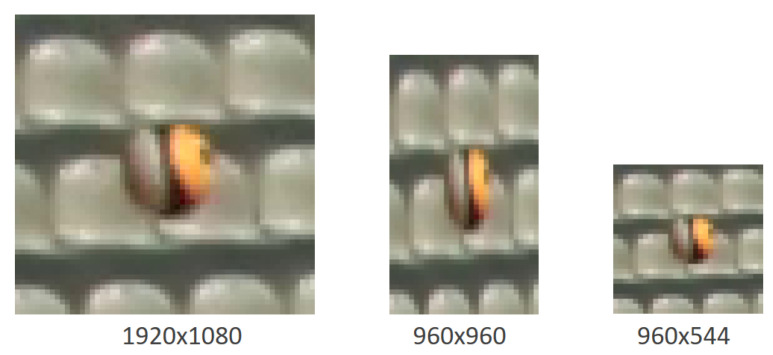
Cut-out of the ball from the original input image in different scales. Downscaling of the original image with a 16:9 aspect ratio to quadratic resolution of 960×960 pixels causes an elongation of the ball. Even though the downscaling to 960×544 pixels does not preserve the original aspect ratio, the characteristic circular shape of the ball is still maintained.

The standard input resolution for YOLOv3 is 416 × 416 pixels. Since the original images usually have higher resolution, they need to be down-sampled to an input size of 416 × 416 prior to the input into the network. With regard to the task of ball detection, this downsampling procedure has two major disadvantages. First of all, fine-grained visual features that are required for the recognition of small, abstract objects, such as balls, may get lost due to potential subsampling of the image. This effect is further reinforced by the fact that for the standard YOLOv3, often equal aspect ratios are used for the network input. Often times, sports footage has an aspect ratio of 16:9, usually Full-HD or even 4K resolution. Resizing to a squared format causes an elongation of objects in the image which results in a change in the characteristic shape of balls in particular and contributes to further distortion of visual characteristics of balls (see Figure 3). If the network is trained on these resized images, the filters are adjusted to the distorted image content. Even though YOLOv3 is invariant to the size of the input image, objects should have a similar distortion during inference as during training to not risk a loss of detection performance.

We try to preserve as much fine-grained visual information as possible on both image axes by training our networks on rectangular input images with limited downsampling factor. For Full-HD video footage in our custom dataset, we observed that an input size of 960×544 pixels preserves most of the important image features on both image axes for the subsequent detection steps. By downscaling the input image resolution to 960×544 pixels, we obtain an aspect ratio of 16:9.1, thereby practically preserving the original input ratio and meeting the requirement that the image dimensions should be a multiple of 32. In this way, we reduce image distortion due to previous downscaling of the image to a minimum, compared to square input images. Accordingly, our network learns to recognize balls as almost circular objects in the input image. This offers the freedom to slightly change the aspect ratio during inference without having to expect a significant drop in accuracy. Furthermore, it simplifies the application of cropping before the network input, since resizing effects do not need to be considered. Prior cropping of a region of interest can improve the detection performance. Image areas that are prone to increased error detections can be excluded from the observation area for detection. A further essential advantage of rectangular image inputs is the reduction of the computing effort during training and inference. We reduce the number of pixels that need to be processed to less than 57 percent compared to a respective squared image input of 960×960 pixels that preserves the same horizontal resolution.

We do not consider variable input resolutions due to the following reasons: first, we want to preserve the possibility of batch processing for further development. Images in batches can be processed in parallel by the GPU which can significantly speed up the inference process. For batch processing, images of fixed height and width are required. Second, arbitrary scaling and cropping of individual frames might have an effect on the size, position, and appearance of the ball in the input images. If these changes are not considered during training, the detection accuracy may decrease in these situations.

### 3.3. Anchor Boxes

YOLOv3 uses k-means clustering on the bounding boxes in the training dataset in order to find good initial estimates for the anchor boxes. Beyond that, we use our prior knowledge about annotated balls in image data. For the detection of uncovered circular balls, the respective annotated bounding boxes are always square-shaped. Therefore, we limit the aspect ratio of the given anchor boxes to be square-shaped as well. If balls are obscured in an image, the respective bounding box may be rectangular, as the covered parts of the ball are not enclosed in the bounding box. For our internal training dataset, we explicitly exclude these cases from the annotated data. Balls are only annotated, if more than half of the ball is visible and sufficiently visible to place a squared bounding box. The bounding box size depends on the size of the ball in the image. The ball size may vary according to the selected lenses and the specific camera settings that are used to capture the game situation but also according to the distance of the ball to the camera. Considering these different distances and image acquisition conditions, we use three scales of anchor boxes for our training dataset, 30×30, 45×45, and 60×60 relative to the original Full HD image resolution.

### 3.4. Architecture Changes

**Figure 4:**
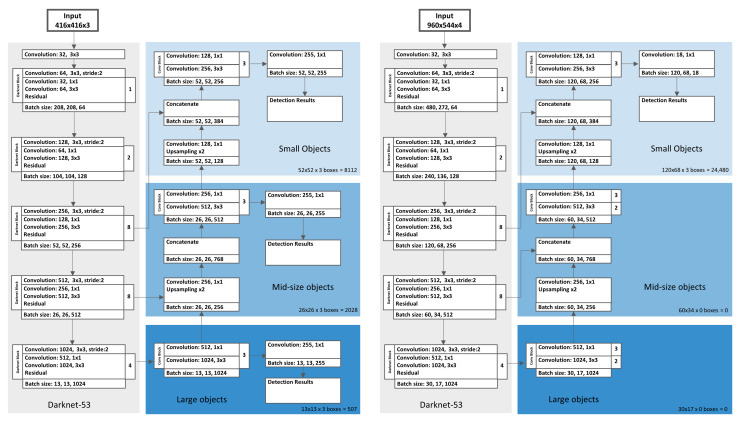
Comparison of the architectures of the standard YOLOv3 with an input resolution of 416 × 416 pixels and the micro model using motion channel and an input resolution of 960 × 544 pixels.

In order to better adapt the YOLOv3 network to our detection task, we apply slight changes to the network architecture (Figure 4).

We use the original Darknet-53 architecture without adjustments and significant changes. By preserving the original architecture, we are able to use the weights and, respectively, the learned feature maps from pre-trained versions of Darknet-53 for transfer learning. All presented models use the weights of the publicly available implementation of Darknet-53 pre-trained on MS COCO dataset [21] as the baseline for training. In order to preserve context information from higher scales, we keep the branches for all three scales. In contrast to the original YOLOv3, we trim the branches for big and mid-size objects, as we do not need their output for the final bounding box predictions.

The dimension of the feature map that predicts the final bounding boxes is defined by downsampling the input image resolutions by the factor 8. For input image dimensions of 960×544 pixels, the resulting grid has a size of 120×68 pixels. Each grid cell predicts three bounding boxes using three square-shaped anchor boxes. The total number of predicted boxes is 120×68×3 = 24,480 boxes. Compared to the standard YOLOv3, our models calculate more bounding boxes, but all boxes are predicted for small objects using a higher resolution grid. Due to the restriction to a single ball class, the adapted network predicts a final output tensor of size 120×68×[3×(4+1+1)] = 146,880. This is a reduction of the output tensor size by 84% compared to the standard YOLOv3, trained on the 80 classes of the COCO dataset, with an output tensor size of 904,995. All networks on which we have implemented the described architectural adaptations to a single scaling level for small objects we refer to as YOLOv3 micro.

### 3.5. Motion Channel

The literature research showed that temporal information can provide important cues for object detection. However, the original YOLOv3 does not make use of temporal information. For the incorporation of this information in the detection pipeline, we aim to integrate the temporal context of the ball movement for detection by providing motion information to the network.

A common approach in video object detection is to provide optical flow fields in order to enhance detection using state-of-the-art object detectors. This either aims to improve detection accuracy by including additional motion information or to increase detection speed as, due to the additional information, not every image has to be processed. Inspired by deep learning-based optical flow methods that output the optical flow field as a color-coded image, we adapt the idea of providing images that encode the motion within multiple contiguous frames of a video sequence. Instead of including an additional neural network for optical flow estimation, we use traditional image differencing approaches in order to keep the architectural complexity low and maintain a simple end-to-end training pipeline.

Numerous traditional approaches for ball detection that are based on conventional image processing algorithms take advantage of difference images in order to investigate the temporal changes between two or more frames. Difference images contain meaningful information about the changes in a sequence of images. Specifically, for the detection of balls, difference images can considerably assist the detection process. In many situations, the ball is the fastest object in the scene and is clearly distinguishable from other moving objects, even when the camera is moving. Especially in front of highly complex backgrounds, on which the ball can not be identified based on the information in a single image only, moving balls show characteristic structures in the respective difference images.

Instead of changing the overall architecture of our detection network, in order to include the temporal dimension, we change the input information provided to the network by including difference images into the network input. To keep the additional processing effort during training and inference low, we do not calculate the differences per channel on the original RGB images. Instead, we apply the image differencing algorithm to the HSV representation of the current input image and its predecessor. We provide only the value channel of the resulting HSV image to the motion channel and discard the hue and saturation channel. Multiple experiments showed that the integration of these two channels did not lead to a noticeable advantage for detection, but increased the computational workload during training and inference. Therefore, we can limit the complementary input to the detection network, in addition to the three color channels of the RGB input image, to a single channel. We refer to this fourth input channel as motion channel.

Image differencing algorithms often take the difference between two images and apply thresholding in order to identify the areas with a significant change in image information. We do not apply any kind of thresholding and leave the selection of relevant information for detection to the network. The HSV difference images, which we refer to as motion frames, are calculated according to the following equation:

(1) $$m_{t}=\left(1-\alpha \right)\cdot 2\cdot \left(HSV\left(f_{t-1}\right)-HSV\left(f_{t}\right)\right)+\alpha \cdot \left(m_{t-1}-128\right)+128$$

where $m_{t}$ denotes the motion frame for an input frame $f_{t}$ at the specific time *t*. The variables $f_{t-1}$ and $m_{t-1}$ denote the previous input frame and the previously calculated motion frame, respectively. The variable α represents the motion fade ratio.

The HSV representation of a frame is calculated according to the following procedure:

V←max(R,G,B)

$$S\leftarrow \left\{\begin{array}{cc} \frac{V-min(R,G,B)}{V} & \mathrm{if}\;V\neq 0\\ 0 & \mathrm{otherwise} \end{array}\right.$$

$$H\leftarrow \left\{\begin{array}{cc} 60(G-B)/\left(V-min(R,G,B)\right) & \mathrm{if}\;V=R\\ 120+60(B-R)/\left(V-min(R,G,B)\right) & \mathrm{if}\;V=G\\ 240+60(R-G)/\left(V-min(R,G,B)\right) & \mathrm{if}\;V=B \end{array}\right.$$

Image areas without changes are grey, image areas that show an decreased brightness compared to previous images appear brighter, and image areas with increased pixel intensity appear darker in the resulting difference image.

For fast moving balls, the positional change of the ball between two consecutive frames is sufficiently large to generate characteristic structures in the difference image. For slower moving balls showing merely slight displacements between two frames, however, it may be beneficial to consider longer image sequences. We therefore apply the motion fade ratio α that indicates the influence of previous frames to the current motion frame calculation. Based on this ratio, we calculate a weighted average of the current difference image and the previous motion frame (Equation (1)). Accordingly, the influence of earlier motions fades over time.

**Figure 5:**
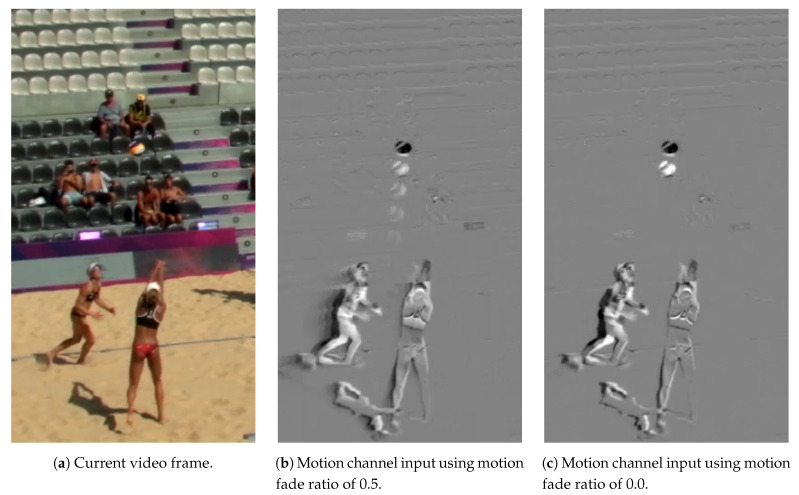
Examples of calculated motion channel inputs for a given input frame using different motion fade ratios.

Figure 5 shows the influence of different motion fade ratios on the input of the motion channel. For the considered video material for beach volleyball, we use a motion fade ratio of 0.0 as the captured balls usually move fast enough between two consecutive images to create characteristic structures in the respective difference image. For other applications where the object displacement between two consecutive frames is not high enough, either due to slower movements or due to higher frame rates, larger motion fade ratios may have a positive effect on the detection performance. Furthermore, larger motion fade ratio values can help to compensate a relative movement of the camera.

Before each inference step, we calculate the respective motion image for each frame and insert the value channel of the motion image into the original frame as an additional channel. Accordingly, we adapt the network input to accept 4-channel input images. The original Darknet-53 architecture itself is unchanged except for the input layer. The feature extractor needs to consider an additional source of information during training and inference.

### 3.6. Data Augmentation

**Figure 6:**
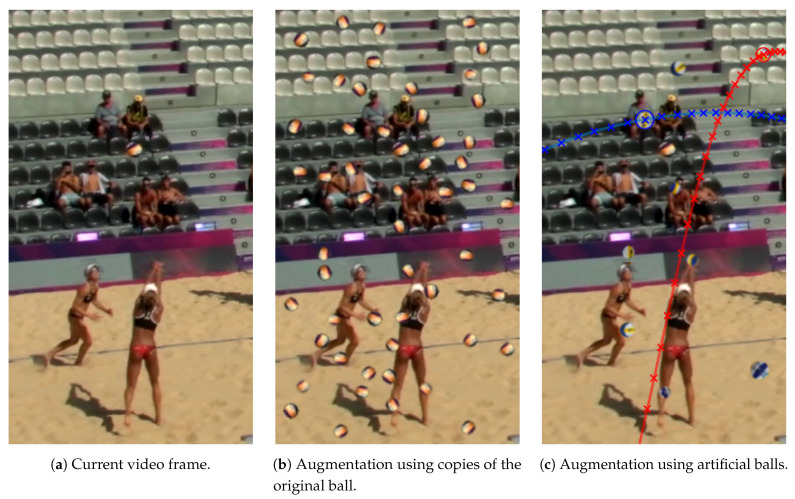
Different augmentation strategies for a given input frame. (**b**) shows the augmentation using multiple copies of the original ball (as used for static augmentation). (**c**) shows the augmentation using previously captured and randomly modified samples of beach volleyball balls (as used for motion augmentation). The red and blue trajectories visualize the simulated movement of two exemplary artificial balls over multiple frames.

In a first step, we focus on the objective to increase the expected intersection between annotations in the training data and predicted bounding boxes during training by applying data augmentation prior to the training process. We achieve this increase by copying labeled balls from the original image and inserting a large number of copies of the ball back into this image. These copies are spread over the entire image in order to account for different possible backgrounds in the image (Figure 6b). The balls are cropped using the inscribed circle (incircle) of the annotated bounding boxes. If the annotations are not square-shaped, we normalize the bounding box to a squared shape by extending the small side of the rectangle until the aspect ratio is equal, while preserving the original center point of the annotated bounding box. To simulate realistic conditions, we blur the edges of the cropped circular patches in order to remove hard cutting edges and to emulate depth perception. Thus, the snippets blend in better into their new background and the network does not learn to recognize synthetically generated, hard edges that are not present in the original image data. Prior to inserting these copies into the image, we apply image transformation to the extracted ball patches in order to simulate different possible conditions. The cropped ball snippets are rotated randomly around their center. To simulate different lighting conditions, the brightness of the snippets is varied randomly by converting the RGB color snippets into their HSV representation and varying the respective brightness value. Depending on the probability of motion blurring in the relevant image/video sequences, we recommend to blur a subset of the ball duplicates by applying Gaussian blur to the snippets. We refer to this data augmentation method as static augmentation.

Especially for extremely small balls, we observed that the possibilities for these image transformation steps are very restricted since excessive transformation steps often lead to unnatural artifacts for low resolution objects. Furthermore, it is often difficult to cut out the original balls precisely without any cutting artifacts from the original image due motion blur or other blurring and occlusion effects.

Therefore, we introduce a second approach for ball data augmentation. We use previously taken photos of the official game ball for beach volleyball, captured from different perspectives, as the base for augmentation. We cut out the ball from these images, apply the mentioned transformations, and finally insert the resulting “artificial” balls into the original image (Figure 6c). Since these previously taken photos are available in higher resolution and the balls can be captured under predefined conditions, the cutting process is more straightforward and the options for transformation are more diverse compared to the low-resolution balls cut from the original images. Therefore, we extend this approach by a temporal component and simulate ball motion across multiple frames. If only individual frames without any temporal context are considered for augmentation, there is a risk of unnatural overlapping and artifacts when calculating the difference images for the motion channel. Accordingly, we simulate the movement of balls across multiple contiguous frames and insert balls according to natural trajectories into the images. We imitate two types of trajectories to account for the characteristics of the ball movements in beach volleyball: First, we simulate the movement of the ball during pass situations (digging, setting). Second, we simulate fast, almost straight trajectories for fast spikes or serves. We refer to this approach as motion augmentation.

For sports with no uniform or predetermined ball design, for example handball, data augmentation can help to improve the generalization ability of the ball detection model. For sports with changing ball designs, we enrich the training data by inserting different ball models. These models do not need to be present in the original data but can be taken from example images instead (for example annotated sports balls from MS COCO dataset [14]). We use different types of balls in the image, in order to train the detector on different models/types of balls and avoid overfitting of the detector on specific balls that are over-represented in the original training data.

### 3.7. Speed Optimization

In order to use the YOLOv3 micro model architecture for real-time analyses, we optimize the detection models for higher inference speeds using NVIDIA TensorRT [45]. Since not all required operations in our custom detection network implementation are supported in the utilized version of TensorRT, we are forced to split the detection model for inference. YOLOv3 applies thresholding and Non-Maximum Suppression (NMS) to remove duplicated bounding boxes with lower confidences. We split off this post-processing step so that we get a two-part inference model. The first network part, including feature extraction and bounding box prediction, is accelerated using TensorRT. For TensorRT optimization, we use FP32 precision in order to match the evaluated accuracy of the developed Tensorflow models best. In terms of latency, there is still room for improvements by changing the specified precision to FP16 or even INT8.

For the post-processing part, we use TensorFlow Lite [46] for inference. Again, we do not apply further optimization steps, such as quantization, which could further accelerate inference but potentially at the expense of accuracy.

## 4. Experiments

### 4.1. Datasets

We evaluate the performance of our detection models on the detection of beach volleyball balls in images captured at professional beach volleyball competitions. We installed cameras around the field of play at three professional venues during competition. We use the data of two venues to train our detection models and evaluate their detection quality and performance on data captured at the third venue. In order to maximize the diversity in the training data, we captured images from different perspectives and under different conditions, such as changing lighting conditions, varying backgrounds, and different recording procedures, such as varying camera settings. In all recorded sequences, the same ball model is used.

The training data set includes 10,363 samples. Each sample consists of an image that captures a real game situation. We manually labeled the bounding box around the game ball, if more than 50 percent of the ball surface was visible in the image. Balls other than the currently used game ball, for example spare balls outside the field of play, were not considered during data annotation. For each sample, we further employed the sequence of the previous images of the captured game situation in order to calculate the current motion frame, which is passed to the motion channel during training. The side lengths of the square-shaped annotated ball bounding boxes in the training data set range from at minimum 10 pixels up to 52 pixels, depending on the distance of the balls from the statically installed cameras. The average bounding box size in the training data set is 22×22 pixels in relation to the 1920×1080 pixels resolution of the original images.

The test data set includes 2192 samples, recorded at the third venue. The images were taken from ten different game situations which were recorded in ten individual continuous video sequences. The different game situations show clear differences in the recording conditions, including different athletes, changing situations in the spectator area of the arena as well as different times of day and, thus, lighting conditions during data acquisition. The average size of the annotated boxes in the test data set is 21×21 pixels.

### 4.2. Models

To evaluate the influence of the presented adaptions of the network architecture and training process compared to the standard YOLOv3 framework, we defined different models that we trained on the custom training data set (Table 1). All architectures share the same feature extraction backend Darknet-53. Therefore, we do not need to train these networks from scratch but instead import the network weights of the Darknet-53 backend, pre-trained on the COCO dataset, from the original YOLOv3 website [21].

Baseline models: The baseline for our development is a re-implementation of the standard YOLOv3 network using TensorFlow 2 in Python, which we refer to as regular YOLOv3. We evaluate the regular YOLOv3 architecture using four different input sizes. For each regular model, we re-train the network on the custom dataset in order to adapt the detection to the different input resolutions. In first instance, we evaluate the baseline model using the standard input resolution of 416×416 pixels. We refer to this model as regular_416 × 416. For evaluation, we analyze two different versions of regular_416 × 416 YOLOv3: First, we import the weights of the original version of YOLOv3 with 416×416 pixels input resolution, pre-trained on the COCO dataset, which includes a class for sports balls already. Secondly, we re-train this network implementation on the custom dataset for beach volleyball.

For the considered beach volleyball dataset, we prefer an input image width of 960 pixels in order to capture all essential image information in sufficient resolution. The standard YOLOv3 is designed to process quadratic input image resolutions. Therefore, we evaluate a second network with an input resolution of 960×960 pixels that we refer to regular_960 × 960. Since the original YOLOv3 paper [21] presents a 608×608 pixels input resolution version in addition to the standard 416×416 pixels resolution, we have further trained a corresponding regular_608 × 608 model to increase the comparability to existing literature and between the different model architectures. To ensure the best possible comparability with the model versions specifically adapted to ball detection, we trained a regular_960 × 544 network accepting a rectangular input resolution of 960×544 pixels which is supposed to be more comparable to our adapted micro YOLOv3 implementation in terms of complexity and the respective input resolution.

Adapted models: All networks on which we have implemented the described architectural adaptations to a single scaling level for small objects we refer to as YOLOv3 micro. All micro variants accept rectangular images with an input resolution of 960×544 pixels. The micro_960 × 544_no_motion model does not consider motion information and only processes RGB input images, comparable to the regular implementations. Model micro_960 × 544 adds a motion channel to account for fast ball motion. The micro_960 × 544_augmentation models use data augmentation for beach volleyball balls during training. We evaluate both augmentation approaches: static augmentation and motion augmentation using different numbers of augmented balls per image. For the micro_960 × 544_augmentation_static_50, 50 artificial balls are inserted for each sample in the training dataset. For micro_960 × 544_augmentation_static_20, 20 copies of the current ball are inserted, respectively. For micro_960 × 544_augmentation_motion_20, micro_960 × 544_augmentation_motion_10, and micro_960 × 544_augmentation_motion_5, we insert 20, 10, and 5 artificial balls into each training sample respectively, following the presented motion augmentation approach.

The model micro_960 × 544_no_motion_augmentation_static_50 does not consider motion information and is trained using static motion augmentation with 50 balls.

### 4.3. Training

We train all model variants using six NVIDIA GTX 1080 Ti GPUs. For training, we use the Adam optimizer with an initial learning rate α=0.001, $\beta_{1}=0.9$, $\beta_{2}=0.999$ and $\epsilon =1\times 10^{-7}$ [47]. All networks are trained on the loss functions introduced in the original YOLO paper [6]. For models with an input width smaller than 960, we use a batch size of 16, otherwise a batch size of 12. In the considered dataset for beach volleyball, the captured balls usually move fast enough between two consecutive images to create characteristic structures in the respective difference image. Therefore, we use a motion fade ratio of 0.0. For both augmentation approaches, static augmentation and motion augmentation, we vary the diameter of the augmented balls between 10 pixels and 52 pixels, representing the dimensions of the samples in the training dataset. For training, we use a threshold score of 0.5 for the objectiveness and a minimum IoU threshold of 0.5 for non-maximum suppression [21].

For the evaluation of the presented model variants, we apply two different types of training strategies:

Initial training: We initialize all model variants using the weights of Darknet-53 pre-trained on the MS COCO dataset. For training, we use the aforementioned hyperparameters. In order achieve the best possible training duration, for each model individually, we apply EarlyStopping. We stop the training process after seven epochs without improvement on the validation dataset.

Refinement training: For the regular_960 × 544 and micro_960 × 544 implementations, we furthermore apply a multi-stage refinement training, in an identical way, for the two network types. In a first step, we train both models using static augmentation with 20 copies of the original ball per frame, following the same strategy as during initial training. Afterwards, we go into a step-by-step refinement phase. In a first refinement run, we augment the training data by inserting 10 additional augmented balls, following the motion augmentation approach. Subsequently, we apply another refinement run using the same approach but decreasing the number of augmented balls per training image to 3 and lower the initial learning rate to 0.0001. Finally, we apply a third refinement training step without any data augmentation at all, still using a learning rate of 0.0001. Our investigations have shown that there is usually no further improvement after the third epoch within a refinement step. Therefore, we stop training after three epochs within a refinement training step, if there is no further improvement of the validation loss, and select the models showing the highest validation accuracy for further refinement training.

We denote all (intermediate) model variants which were trained following the refinement training approach with the suffix _rt indicating “refinement training”, followed by the respective number of augmented balls in this phase.

### 4.4. Evaluation Metrics

We evaluate all models according to the respective training methods based on different metrics: (1) number of true positive detections (TP), false positive detections (FP), and false negatives (FN), (2) precision, (3) recall, (4) F1 score, (5) average precision of class “ball” (AP), (6) averaged intersection over union (IoU) over all true positive detections in the test data set, (7) number of network parameters, (8) inference time in frames per second (FPS), and (9) training time per epoch and sample.

For the calculation of the AP, we use the mean average precision (mAP) criterion defined in the PASCAL VOC 2012 competition [48]. All detection results are sorted by decreasing confidence scores and assigned to ground truth objects. We assign a detection to a ground truth object if the respective IoU≥0.5. Using these assignments, we calculate the precision-recall curve with monotonically decreasing precision. For a specific recall *r*, we set the corresponding precision to the maximum precision achieved for any recall $r^{\prime }>r$. We calculate the AP for the single class ball as the area under the precision-recall curve. The mAP is calculated as the mean of the AP scores over all object classes present in the ground truth data. Since there is only one class in our dataset, AP and mAP provide the same metric. Therefore, we limit our evaluation to the presentation of the AP for the class ball. We separately indicate the IoU in order to better specify the spatial accuracy of detection for a specific model variant.

Following the same approach as for training, we use a threshold score of 0.5 for the objectiveness for inference, in order to define if an object is detected as such or not. The minimum IoU threshold for non-maximum suppression is also set to 0.5.

We run the evaluation without batch processing on a NVIDIA RTX 2080 Ti GPU. All models are evaluated using Python 3.6 and Tensorflow 2.

Table 2 and Table 3 show the results of the different network architectures trained on the custom training dataset for beach volleyball (except for regular_416 × 416_COCO which was not re-trained). The performance is evaluated on data captured at a third, independent venue. No data from this venue is present in the dataset used for training.

## 5. Results

### 5.1. Performance

The regular_416 × 416_COCO model that was merely pre-trained on the COCO dataset does not ensure any reliable detection at all on the given data set. Only in a very few isolated cases the ball is detected and classified correctly as a sports ball. Re-training on the specific footage increases the detection performance significantly. The regular_416 × 416 shows a comparatively high recall (81.0%). In contrast, precision is low due to a high number of false positive detections. With increasing input resolution for the regular implementations, the average precision (AP) increases up to a maximum value of 90.2% for the regular_960 × 960 model. However, as the square input size increases for the regular models, the precision decreases significantly, down to a value of 27.3% for the regular_960 × 960 model. The regular_960 × 960 model has the highest recall of all models but, at the same time, the lowest overall precision. An exception is regular_960 × 544 that shows precision results comparable to the lower resolution regular_416 × 416 model while recall and AP are higher. In general, with increasing input resolution, an improvement of the IoU can be observed within the re-trained regular models.

All micro implementations are evaluated for an input image resolution of 960 × 544 pixels. The model micro_960 × 544_no_motion has an almost identical AP compared to the reference model regular_960 × 544. However, the F1 score of 72.6% is slightly higher than for the regular version, mainly due to increased precision, but with slightly lower recall. In comparison to micro_960 × 544_no_motion with an AP of 84.1%, we added static augmentation with 50 additional balls during training process for model micro_960 × 544_no_motion _augmentation_static_50, which results in an increased AP of 89.7%. Even though AP increased, precision and accordingly F1 score are comparatively low. Compared to the regular models, an increase in IoU to 83.0% can be observed.

The training of a micro model that considers motion information without augmentation during the training process results in a noticeably decreased average precision during test time. The model micro_960 × 544 shows a very low AP of 72.9%. It is conspicuous, however, that F1 score (80.5%) and precision (83.2%) for micro_960 × 544 are significantly higher than for all presented regular implementations.

We applied both presented types of augmentation during initial training. The model variant micro_960 × 544_augmentation_static_20 reaches an AP of 78.1%. An increasing number of inserted balls for static augmentation does not improve the AP significantly. All other evaluated metrics decrease for micro_960 × 544_augmentation _static_50 and micro_960 × 544_augmentation_static_75 compared to micro_960 × 544 _augmentation_static_20. For the micro models trained using motion augmentation, the AP decreases while the number of inserted artificial balls increases, reaching from 74.5% AP for micro_960 × 544_augmentation_motion_5 to 62.7% for micro_960 × 544 _augmentation_motion_20. Generally, the precision for micro models trained using motion augmentation is high, reaching a maximum of 90.2% for micro_960 × 544_augmentation_motion_10.

We observe a significant increase in recall and accordingly F1 score and AP for micro models with motion channel when refinement training is applied. micro_960 × 544 _augmentation_motion_3_rt achieves an AP of 89.0%, almost reaching the maximum AP of 90.2% of the higher-resolution regular_960 × 960. Furthermore, this model achieves the highest IoU of 84.5% across all evaluated models. The highest F1 score (88.9%) can be observed after the first refinement run for model micro_960 × 544_augmentation_motion_10_rt. During the last refinement run without additional augmentation, no further improvement in performance can be measured. To better evaluate the effect of refinement training, we applied the same approach to the regular_960 × 544. Especially after the second refinement run, the precision increases to 79.2% for regular_960 × 544_augmentation_motion_3_rt compared to the same model trained without augmentation. Furthermore, an average IoU of 84.1% is noticeably higher than compared to standard training. Even though regular_960 × 544_rt reaches comparable AP as the baseline model, precision (73.2%) and recall (90.4%) are more balanced and the resulting F1 score (80.9%) is higher.

### 5.2. Speed

With regard to an objective comparison of different detection models, we initially evaluate the inference speed of all model variants using our re-implementation of the standard YOLOv3 network using TensorFlow 2 in Python. Our regular_416 × 416 model achieves around 16 FPS on a RTX 2080 Ti using the standard input resolution of 416×416 pixels. For an input resolution of 960×960 pixels, the inference speed is on average 8.1 FPS. For the regular_960 × 544, we achieve an average frame rate of 11.7 FPS. Our custom implementation micro_960 × 544_motion that processes motion information by the additional motion channel achieves an average inference speed of 12.9 FPS. Without motion channel, the average processing speed even increases to 13.4 FPS.

Even though a frequency of 10 FPS is sufficient to describe the movement of the ball in most cases, game determining situations with high ball speeds cannot be captured and analyzed adequately. With an optimized version of the YOLOv3 micro model with motion channel, we are able to perform the inference with up to 40 FPS. The inference time for the results of the first subnetwork using TensorRT on a NVIDIA RTX 2080 Ti is 0.011 seconds on average. The inference of the post-processing steps using TensorFlow Lite takes about 0.003 seconds on average and, therefore, has little influence on the overall prediction speed. Since the motion information is required as a network input, the difference image calculation must be considered in the overall inference time. With an average time of about 0.011 seconds for the difference image calculation, the overall processing time is 0.025 seconds per frame and thus enables processing in real time.

In addition to the speed increase during inference for the micro models, the time required for model training is decreased. The time required per epoch during training is reduced from 3:23 hours on average for the regular_960 × 544 to an average of 1:10 hours for the micro_960 × 544. The time required for training increases with the number of balls inserted during augmentation.

## 6. Discussion

### 6.1. Effect of Input Resolution

The evaluation of the regular models trained on different image input sizes allows for a good analysis of the correlation between input resolution and detection performance. Generally, YOLOv3 allows for an easy trade-off between speed and accuracy, just by changing the input size, without more complex adaptations of the network architecture. For the purpose of ball detection, the limitations of this easy trade-off are clearly visible: While the processing speed decreases, AP, recall, and IoU increase with increasing input resolutions. In contrast, precision decreases with increasing square-shaped input resolutions as the number of false positive detections increases significantly. The better ratio between precision and recall and accordingly the higher F1 score for the regular_960 × 544 model is a clear indication that nearly preserving the original image aspect ratio for training and inference instead of downscaling to a square-shaped input size preserves fine-grained visual information that is required to distinguish balls from other objects and structures in the input image. The slightly increased IoU for larger input sizes indicates that the predicted bounding boxes better estimate the actual position of the ball in the image when the input resolution is increased.

### 6.2. Effect of Motion Channel

**Figure 7:**
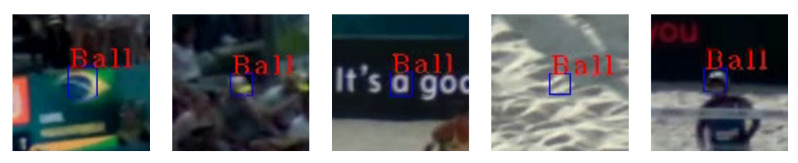
Examples for false positive detections by the reference model regular_960 × 544 that are suppressed by the micro_960 × 544_augmentation_motion_3_rt model using motion information. The examples show similar structures to the ball in the RGB image. However, they do not produce characteristic patterns in the motion channel and are consequently not recognized as balls.

Networks without motion channel perform stably, especially for balls on homogeneous backgrounds. In front of relatively complex backgrounds, which show similar structures to the ball, for example spectator areas or animated perimeter advertising, we identified a high number of false positive detections in the background areas of the scene (Figure 7). Often, these false positive detections are persistent within consecutive frames of a video.

In contrast to micro_960 × 544_no_motion, micro_960 × 544 adds the motion channel to the micro network. The micro_960 × 544 model exhibits significantly reduced AP compared to its non-motion counterpart. The precision is apparently higher due to a significantly lower number of FP. However, the improvements in precision are at the expense of a lowered recall. Accordingly, recall and precision are balanced for models with motion channel: without adaption, these models detect fewer balls but produce significantly fewer false positive detections. The increased precision for models with motion channel shows that the motion information helps the model to better detect relevant objects and to suppress error detections. Consistent false positive detection of static structures in the background, for example heads in the spectators area or circular objects on the perimeter advertising, are minimized. However, the motion information is not yet used to continuously detect balls in consecutive frames without interruptions. We assume that, without augmentation, ball samples including valuable corresponding motion information are underrepresented during training.

### 6.3. Effect of Data Augmentation

The significant improvement in AP between micro_960 × 544_no_motion and micro_960 × 544_no_motion_augmentation_static_50 indicates the positive influence of static augmentation to models that do not consider motion information. Static augmentation seems to improve the feature extraction and detection process on the original RGB input image, leading to better performance during inference. We hypothesize that especially the feature extraction in the Darknet-53 part of the micro models benefits from the increase in training targets per image. The comparison between the models micro_960 × 544, without augmentation, and micro_960 × 544_augmentation_static_20 further shows that the positive effect of static augmentation is also present for models with motion channel. In turn, different analyses showed that an intensive increase of augmented balls for static augmentation can not improve the detection further.

Besides static augmentation, we further evaluated the effect of motion augmentation on the network performance. Comparable to static augmentation, the performance of models trained with an increased number of inserted balls using motion augmentation decreases. For regular models and micro models without motion channel, the recall is consistently high while precision is relatively low. For micro models that are trained without augmentation or trained using static augmentation (micro_960 × 544_augmentation_static_20 and micro_960 × 544_augmentation_static_75), precision and recall are balanced. However, micro models trained using motion augmentation tend to show higher precision while recall decreases. As motion augmentation further increases precision, this augmentation approach seems to additionally intensify the influence of motion information to the detection process. However, this is done at the expense of significantly decreased recall. Models using a motion channel tend to be highly sensitive to an exclusive application of motion augmentation. We assume that the inserted artificial balls do not sufficiently simulate the visual features of the original balls in the image data. Accordingly, the feature extraction process is overadapted to the artificially created balls. To achieve the necessary balance between the two types of data augmentation, we apply refinement training.

Compared to the static augmentation approach, where the modified balls are distributed randomly in each image, we were able to significantly improve the detection quality especially at the reversal points of the ball parabolas, as both fast and slow ball movements are included in the augmented training data when applying motion augmentation.

### 6.4. Effect of Refinement Training

Refinement training significantly improves performance on micro models using motion information. The first training phase, using static augmentation, enhances the feature extraction based on visual features in the original RGB images. The following refinement steps increase the influence of valuable motion information while maintaining the balance between static features in the image and motion information. Using refinement training, the performance of micro models including motion can be significantly increased compared to the regular_960 × 544 baseline model.

### 6.5. Effect of Architectural Changes

The preservation of the original Darknet-53 architecture offers two major advantages: First, we are able to use the weights and respectively, the learned feature maps from pre-trained versions of Darknet-53 for transfer learning. This significantly reduces the duration of the training process compared to training the models from scratch. Furthermore, we observed that training a fully customized YOLOv3-based network for ball detection from scratch is a fairly complex task that requires to deal with significant model instability during training. The training stability gets significantly improved by loading the pre-trained Darknet-53 weights before training. A possible increase in speed by modifying the original Darknet backend architecture does not outweigh the resulting improvements in training stability and speed.

We analyzed the detection accuracy of the standard YOLOv3 model trained on the MS COCO dataset on our training dataset. We observed that a correct detection was limited to rare individual cases. If a ball was detected correctly as a sports ball, the respective bounding box was predicted at the 52×52 feature map in the branch that is responsible for the detection of small objects. In a first attempt, we fully removed the branches for large and medium sized objects and downsized the feature extractor network by disabling all layers after the third Darknet Block that do not directly contribute to the detection in the small objects branch. After training on our internal dataset, we observed a significant increase in false positives’ detections. We assume that this behavior was caused by the loss of additional context information generated in the removed branches for big and mid-size objects. The detection network is able to use the fine-grained features from earlier layers in Darknet in order to detect the ball correctly. However, there is missing information on high scale features and semantic information that can be used to suppress error detections. In order to preserve context information from higher scales, we keep the branches for all three scales but trim the branches for big and mid-size objects and thus exclude them from the calculation of the detection results.

During inference, the adjustments in the architecture to limit the detection calculation to the small object scale only have primarily influenced the detection speed of the network. Comparing regular_960 × 544 and micro_960 × 544_no_motion, AP and IoU do not show any major deviations. Precision and F1 are slightly increased for the micro model, which is most likely due to the adjustments of the anchor box sizes. On the other hand, micro_960 × 544 with a total of 61,576,342 network parameters has about 5,931,780 parameters fewer than the corresponding regular implementation. This results in the increase of inference speed without a loss in performance. In addition, the time required per epoch during training is reduced for the micro model versions. This acceleration is mainly achieved by the elimination of the predicted boxes in the mid-size and large object scale branches compared to the original regular implementations. Accordingly, only the remaining predicted boxes in the small object branch are considered during training, for example for loss calculation and parameter update.

### 6.6. Final Model Selection

**Figure 8:**
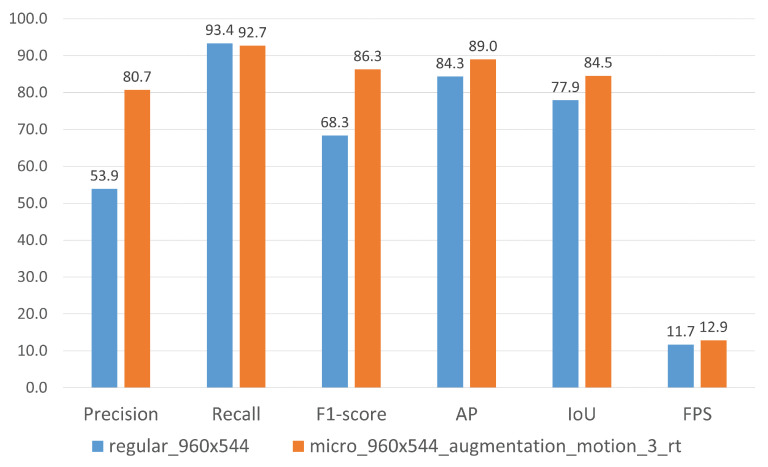
Comparison between the reference model regular_960 × 544 and the finally selected micro model micro_960 × 544_augmentation_motion_3_rt with motion channel. Due to the suppression of false positive detections, a significantly higher recall and consequently a higher F1-score is achieved. At the same time, the processing speed is increased by more than 10%.

Models show different characteristics at different stages of the refinement training process. The model micro_960 × 544_augmentation_motion_3_rt shows the highest IoU over all evaluated models and the highest AP within the refinement training process of the micro model. In turn, micro_960 × 544_augmentation_motion_10_rt exhibits the highest F1 score over all evaluated models. The selection of the final model primarily depends on the intended purpose of the detection model and the requirements of the specific use case. We select micro_960 × 544_augmentation_motion_3_rt as the final detection model as it offers the highest number of TP detections within the refinement training process and has the highest IoU. Figure 8 shows the comparison of the selected final model with the baseline model regular_960 × 544.

## 7. Summary and Conclusions

In this paper, we propose specific adjustments to the original YOLOv3 network architecture and training process with the goal to improve both the detection accuracy and speed, for the specific task of sports ball detection in sports video footage. We apply architectural changes to the original YOLOv3 network that lead to an enhanced detector for small objects which we refer to as micro YOLOv3. To account for fast movements of the detection targets, we incorporate motion information into the detection process by inserting an additional motion channel to the detection network. Furthermore, we present a multi-stage refinement training approach, based on multimodal data augmentation, in order to adapt the training process to the architectural changes. We provide an extensive analysis of the effect of the proposed changes regarding various performance metrics.

Our final model outperforms the re-trained YOLOv3 baseline architecture, both in terms of detection accuracy and speed on a custom dataset for beach volleyball ball detection. Therefore, the presented approach provides an enhancement of the classical trade-off between speed and accuracy for YOLOv3 models. Both detection speed and accuracy are increased simultaneously using prior knowledge about the specific detection task. We demonstrate that our final model architecture can be used for real-time applications with up to 40 FPS using hardware-based optimization.

We focused the development and evaluation of the introduced detection approach on the specific task of ball detection in beach volleyball for the custom dataset available to us. In order to transfer the detection approach to other sports, the proposed enhancements need to be tuned to the characteristics of the intended use cases. This concerns especially the adaption of the data augmentation procedure to account for different ball designs and different characteristic motions of balls and accordingly the adjustment of the influence of the motion channel. Furthermore, we limited our evaluation to video data captured by static cameras. The influence of camera motion has to be evaluated on a case-by-case basis.

Even though the presented approach was designed for the detection of sports balls in particular, the introduced adaptations can be transferred to other use cases where small, fast moving objects need to be detected in video footage.

## 8. Future Work

We demonstrate that the application of refinement training that incorporates both static augmentation and motion augmentation supports the integration of motion information into the detection process without decreasing the influence of static image content. However, a multi-stage training process is difficult to handle. Therefore, we will further investigate the possibilities to integrate both augmentation methods into a single end-to-end training process.

We used YOLOv3 as the basis for the presented adaptions. To date, YOLOv3 is one of the state-of-the-art models for real-time object detection. However, in 2020, three major versions of YOLO, YOLOv4 [49], YOLOv5, and PP-YOLO [50], with improved performance compared to YOLOv3, have been released. YOLOv4 is still based on Darknet-53 but introduces two sets of improvements: BoF (bag of freebies) that enhances detection accuracy without increasing the inference time (yet at the expense of increased training time) and BoS (bag of specials) that slightly increases the inference costs but significantly improves the detection accuracy. AP is increased by 10% and FPS increased by 12% compared to YOLOv3. We will take these improvements into consideration as we continue to work on our presented detection approaches.

Even though the use of traditional image processing techniques to compute the motion information as additional network input has several advantages, we will investigate the use of optical flow methods in the future. Provided these models can be incorporated into an end-to-end training pipeline without significantly increasing the amount of training data required, they can be a source for improved detection performance.

Due to the limitations of the available dataset, the transferability to other sports could only be considered to a limited extent so far. Accordingly, we will evaluate the suitability of the presented detection approach for other sports such as volleyball or handball in the next steps. Our goal is to develop a universal ball detection model that can be applied across different sports and for different image acquisition conditions without further adaption.

An important factor in the detection process involving motion information is the movement of the camera during recording. We will further investigate the influence of camera motion on the detection process. We plan to establish a camera motion compensation that recognizes the movement of the camera and excludes the resulting motion information from the ball detection.

## Figures and Tables

**Table 1:** Overview of all evaluated model variants, including the respective input image size and the applied augmentation strategy (static augmentation or motion augmentation). Column “Motion” indicates whether motion information is considered by the model. We denote all (intermediate) model variants which are trained following the refinement training approach with the suffix _rt indicating “refinement training”.

Model	Augmentation	Motion	Input Width	Input Height
regular_416 × 416_COCO			416	416
regular_416 × 416			416	416
regular_608 × 608			608	608
regular_960 × 960			960	960
regular_960 × 544			960	544
				
micro_960 × 544_augmentation_static_75	static	√	960	544
micro_960 × 544_augmentation_static_50	static	√	960	544
micro_960 × 544_augmentation_static_20	static	√	960	544
				
micro_960 × 544_augmentation_motion_5	motion	√	960	544
micro_960 × 544_augmentation_motion_10	motion	√	960	544
micro_960 × 544_augmentation_motion_20	motion	√	960	544
				
micro_960 × 544		√	960	544
micro_960 × 544_no_motion			960	544
micro_960 × 544_no_motion_augmentation_50	static		960	544
				
micro_960 × 544_augmentation_motion_10_rt	motion	√	960	544
micro_960 × 544_augmentation_motion_3_rt	motion	√	960	544
micro_960 × 544_rt		√	960	544
				
regular_960 × 544_augmentation_motion_10_rt	motion		960	544
regular_960 × 544_augmentation_motion_3_rt	motion		960	544
regular_960 × 544_rt			960	544

**Table 2:** Comparison of training and inference speed, in relation to the network input size and the number of network parameters, of the different model variants adapted on the custom dataset for beach volleyball balls.

Model	Input Width	Input Height	Input Pixel	Trainable Parameters	Non-Trainable Parameters	Parameters	FPS	Training Time (Epoch)	Training Time (Sample)
regular_416 × 416_COCO	416	416	173,056	61,523,734	52,608	61,576,342	16.2		
regular_416 × 416	416	416	173,056	61,523,734	52,608	61,576,342	16.2	3:12	5.267
regular_608 × 608	608	608	369,664	61,523,734	52,608	61,576,342	12.9	3:14	5.325
regular_960 × 960	960	960	921,600	61,523,734	52,608	61,576,342	8.1	3:21	5.525
regular_960 × 544	960	544	522,240	61,523,734	52,608	61,576,342	11.7	3:23	5.584
									
micro_960 × 544_augmentation_static_75	960	544	522,240	55,595,026	49,536	55,644,562	12.9	2:59	4.926
micro_960 × 544_augmentation_static_50	960	544	522,240	55,595,026	49,536	55,644,562	12.9	2:22	3.92
micro_960 × 544_augmentation_static_20	960	544	522,240	55,595,026	49,536	55,644,562	12.9	1:37	2.672
									
micro_960 × 544_augmentation_motion_5	960	544	522,240	55,595,026	49,536	55,644,562	12.9	1:18	2.145
micro_960 × 544_augmentation_motion_10	960	544	522,240	55,595,026	49,536	55,644,562	12.9	1:29	2.457
micro_960 × 544_augmentation_motion_20	960	544	522,240	55,595,026	49,536	55,644,562	12.9	1:36	2.631
									
micro_960 × 544	960	544	522,240	55,595,026	49,536	55,644,562	12.9	1:10	1.937
micro_960 × 544_no_motion	960	544	522,240	55,594,738	49,536	55,644,274	13.4	1:11	1.946
micro_960 × 544_no_motion_augmentation_50	960	544	522,240	55,594,738	49,536	55,644,274	13.4	1:29	2.459
									
micro_960 × 544_augmentation_motion_10_rt	960	544	522,240	55,595,026	49,536	55,644,562	12.9	1:29	2.460
micro_960 × 544_augmentation_motion_3_rt	960	544	522,240	55,595,026	49,536	55,644,562	12.9	1:15	2.059
micro_960 × 544_rt	960	544	522,240	55,595,026	49,536	55,644,562	12.9	1:12	1.974
									
regular_960 × 544_augmentation_motion_10_rt	960	544	522,240	61,523,734	52,608	61,576,342	11.7	3:32	5.830
regular_960 × 544_augmentation_motion_3_rt	960	544	522,240	61,523,734	52,608	61,576,342	11.7	3:26	5.666
regular_960 × 544_rt	960	544	522,240	61,523,734	52,608	61,576,342	11.7	3:29	5.747

**Table 3:** Performance comparison of the different model variants adapted on the custom dataset for beach volleyballs. Underlined values indicate the best results for the respective evaluation metric.

Model	GT	TP	FP	FN	Precision	Recall	F1	AP	IoU
regular_416 × 416_COCO	2189	11	2	2178	84.6	0.5	1.0	0.5	63.6
regular_416 × 416	2189	1774	1679	415	51.4	81.0	62.9	73.9	71.4
regular_608 × 608	2189	2085	3555	104	37.0	95.2	53.3	82.7	76.6
regular_960 × 960	2189	2157	5752	32	27.3	**98.5**	42.7	**90.2**	79.2
regular_960 × 544	2189	2044	1748	145	53.9	93.4	68.3	84.3	77.9
									
micro_960 × 544_augmentation_static_75	2189	1774	458	415	79.5	81.0	80.3	76.0	80.0
micro_960 × 544_augmentation_static_50	2189	1789	1488	400	54.6	81.7	65.5	69.5	76.9
micro_960 × 544_augmentation_static_20	2189	1803	370	386	83.0	82.4	82.7	78.1	79.5
									
micro_960 × 544_augmentation_motion_5	2189	1734	364	455	82.7	79.2	80.9	74.5	79.6
micro_960 × 544_augmentation_motion_10	2189	1451	158	738	**90.2**	66.3	76.4	63.7	79.1
micro_960 × 544_augmentation_motion_20	2189	1452	241	737	85.8	66.3	74.8	62.7	77.6
									
micro_960 × 544	2189	1704	343	485	83.2	77.8	80.5	72.9	78.9
micro_960 × 544_no_motion	2189	2001	1319	188	60.3	91.4	72.6	84.1	79.3
micro_960 × 544_no_motion_augmentation_50	2189	2111	2528	78	45.5	96.4	61.8	89.7	83.0
									
micro_960 × 544_augmentation_motion_10_rt	2189	1966	270	223	87.9	89.8	**88.9**	86.2	83.8
micro_960 × 544_augmentation_motion_3_rt	2189	2029	485	160	80.7	92.7	86.3	89.0	**84.5**
micro_960 × 544_rt	2189	2021	482	168	80.7	92.3	86.1	88.3	83.9
									
regular_960 × 544_augmentation_motion_10_rt	2189	2062	1706	127	54.7	94.2	69.2	87.0	83.3
regular_960 × 544_augmentation_motion_3_rt	2189	1926	507	263	79.2	88.0	83.3	82.7	84.1
regular_960 × 544_rt	2189	1978	725	211	73.2	90.4	80.9	84.4	83.8

## Data Availability

The data presented in this study are available on request from the corresponding author. The data are not publicly available due to data protection regulations.

## Abbreviations

The following abbreviations are used in this manuscript:

AP	Average Precision
BoF	Bag of Freebies
BoS	Bag of Specials
FCN	Fully Convolutional Network
CNN	Convolutional Neural Networks
FN	False Negatives
FP	False Positives
FPS	Frames per Second
GT	Ground Truth
HSV	Hue, Saturation, Value
IoU	Intersection over Union
mAP	Mean Average Precision
NMS	Non-Maximum Suppression
RGB	Red, Green, Blue
TP	True Positives

## References

[1] Link D. (2018). Sports Analytics. Ger. J. Exerc. Sport Res..

[2] Thomas G., Gade R., Moeslund T.B., Carr P., Hilton A. (2017). Computer Vision for Sports: Current Applications and Research Topics. Comput. Vis. Image Underst..

[3] Burić M., Pobar M., Ivašić-Kos M. Object Detection in Sports Videos. Proceedings of the 2018 41st International Convention on Information and Communication Technology, Electronics and Microelectronics (MIPRO).

[4] Tong K., Wu Y., Zhou F. (2020). Recent advances in small object detection based on deep learning: A review. Image Vis. Comput..

[5] Kamble P.R., Keskar A.G., Bhurchandi K.M. (2019). Ball Tracking in Sports: A Survey. Artif. Intell. Rev..

[6] Redmon J., Divvala S., Girshick R., Farhadi A. You Only Look Once: Unified, Real-Time Object Detection. Proceedings of the IEEE Conference on Computer Vision and Pattern Recognition (CVPR).

[7] Zhao Z.Q., Zheng P., Xu S.t., Wu X. (2019). Object Detection with Deep Learning: A Review. IEEE Trans. Neural Netw. Learn. Syst..

[8] Lowe D.G. (2004). Distinctive Image Features from Scale-Invariant Keypoints. Int. J. Comput. Vis..

[9] Dalal N., Triggs B. Histograms of Oriented Gradients for Human Detection. Proceedings of the 2005 IEEE Computer Society Conference on Computer Vision and Pattern Recognition (CVPR’05).

[10] Lienhart R., Maydt J. An Extended Set of Haar-like Features for Rapid Object Detection. Proceedings of the International Conference on Image Processing.

[11] Cortes C., Vapnik V. (1995). Support Vector Machine. Mach. Learn..

[12] Jones M., Viola P. (2003). Fast Multi-view Face Detection. Mitsubishi Electric Research Lab TR-20003-96.

[13] Deng J., Dong W., Socher R., Li L.J., Li K., Fei-Fei L. ImageNet: A Large-Scale Hierarchical Image Database. Proceedings of the 2009 IEEE Conference on Computer Vision and Pattern Recognition.

[14] Lin T.Y., Maire M., Belongie S., Hays J., Perona P., Ramanan D., Dollár P., Zitnick C.L. Microsoft COCO: Common Objects in Context. Proceedings of the European Conference on Computer Vision.

[15] Soviany P., Ionescu R.T. Optimizing the Trade-Off between Single-Stage and Two-Stage Deep Object Detectors using Image Difficulty Prediction. Proceedings of the 2018 20th International Symposium on Symbolic and Numeric Algorithms for Scientific Computing (SYNASC).

[16] Ren S., He K., Girshick R., Sun J. (2015). Faster R-CNN: Towards Real-Time Object Detection with Region Proposal Networks. arXiv.

[17] He K., Gkioxari G., Dollár P., Girshick R. Mask R-CNN. Proceedings of the IEEE International Conference on Computer Vision (ICCV).

[18] Dai J., Li Y., He K., Sun J. (2016). R-FCN: Object Detection via Region-based Fully Convolutional Networks. arXiv.

[19] Liu W., Anguelov D., Erhan D., Szegedy C., Reed S., Fu C.Y., Berg A.C. SSD: Single Shot MultiBox Detector. Proceedings of the European Conference on Computer Vision.

[20] Redmon J., Farhadi A. YOLO9000: Better, Faster, Stronger. Proceedings of the IEEE Conference on Computer Vision and Pattern Recognition (CVPR).

[21] Redmon J., Farhadi A. (2018). YOLOv3: An Incremental Improvement. arXiv.

[22] Huang J., Rathod V., Sun C., Zhu M., Korattikara A., Fathi A., Fischer I., Wojna Z., Song Y., Guadarrama S. Speed/Accuracy Trade-Offs for Modern Convolutional Object Detectors. Proceedings of the IEEE Conference on Computer Vision and Pattern Recognition (CVPR).

[23] Buric M., Pobar M., Ivasic-Kos M. Ball Detection using YOLO and Mask R-CNN. Proceedings of the 2018 International Conference on Computational Science and Computational Intelligence (CSCI).

[24] Burić M., Pobar M., Ivašić-Kos M. Adapting YOLO Network for Ball and Player Detection. Proceedings of the 8th International Conference on Pattern Recognition Applications and Methods (ICPRAM 2019).

[25] Kisantal M., Wojna Z., Murawski J., Naruniec J., Cho K. (2019). Augmentation for Small Object Detection. arXiv.

[26] Montserrat D.M., Lin Q., Allebach J., Delp E.J. (2017). Training Object Detection And Recognition CNN Models Using Data Augmentation. Electron. Imaging.

[27] Weng L. (2018). Object Detection Part 4: Fast Detection Models. lilianweng.github.io/lil-log.

[28] He K., Zhang X., Ren S., Sun J. Deep Residual Learning for Image Recognition. Proceedings of the IEEE Conference on Computer Vision and Pattern Recognition (CVPR).

[29] Lin T.Y., Dollár P., Girshick R., He K., Hariharan B., Belongie S. Feature Pyramid Networks for Object Detection. Proceedings of the IEEE Conference on Computer Vision and Pattern Recognition (CVPR).

[30] Russakovsky O., Deng J., Su H., Krause J., Satheesh S., Ma S., Huang Z., Karpathy A., Khosla A., Bernstein M. (2015). ImageNet Large Scale Visual Recognition Challenge. Int. J. Comput. Vis. IJCV.

[31] Han W., Khorrami P., Paine T.L., Ramachandran P., Babaeizadeh M., Shi H., Li J., Yan S., Huang T.S. (2016). Seq-NMS for Video Object Detection. arXiv.

[32] Hou R., Chen C., Shah M. (2017). An End-to-end 3D Convolutional Neural Network for Action Detection and Segmentation in Videos. arXiv.

[33] Hara K., Kataoka H., Satoh Y. Learning Spatio-Temporal Features with 3D Residual Networks for Action Recognition. Proceedings of the IEEE International Conference on Computer Vision (ICCV).

[34] Ji S., Xu W., Yang M., Yu K. (2012). 3D Convolutional Neural Networks for Human Action Recognition. IEEE Trans. Pattern Anal. Mach. Intell..

[35] Xiao F., Jae Lee Y. Video Object Detection with an Aligned Spatial-Temporal Memory. Proceedings of the European Conference on Computer Vision (ECCV).

[36] Liu M., Zhu M. Mobile Video Object Detection with Temporally-Aware Feature Maps. Proceedings of the IEEE Conference on Computer Vision and Pattern Recognition.

[37] Baker S., Matthews I. (2004). Lucas-Kanade 20 Years On: A Unifying Framework. Int. J. Comput. Vis..

[38] Dosovitskiy A., Fischer P., Ilg E., Hausser P., Hazirbas C., Golkov V., Van Der Smagt P., Cremers D., Brox T. FlowNet: Learning Optical Flow with Convolutional Networks. Proceedings of the IEEE International Conference on Computer Vision (ICCV).

[39] Ilg E., Mayer N., Saikia T., Keuper M., Dosovitskiy A., Brox T. FlowNet 2.0: Evolution of Optical Flow Estimation with Deep Networks. Proceedings of the IEEE Conference on Computer Vision and Pattern Recognition (CVPR).

[40] Ranjan A., Black M.J. Optical Flow Estimation Using a Spatial Pyramid Network. Proceedings of the IEEE Conference on Computer Vision and Pattern Recognition (CVPR).

[41] Sun D., Yang X., Liu M.Y., Kautz J. (2019). Models Matter, So Does Training: An Empirical Study of CNNs for Optical Flow Estimation. IEEE Trans. Pattern Anal. Mach. Intell..

[42] Zhu X., Xiong Y., Dai J., Yuan L., Wei Y. Deep Feature Flow for Video Recognition. Proceedings of the IEEE Conference on Computer Vision and Pattern Recognition (CVPR).

[43] Zhu X., Wang Y., Dai J., Yuan L., Wei Y. Flow-Guided Feature Aggregation for Video Object Detection. Proceedings of the IEEE International Conference on Computer Vision.

[44] Zhu X., Dai J., Yuan L., Wei Y. Towards High Performance Video Object Detection. Proceedings of the IEEE Conference on Computer Vision and Pattern Recognition (CVPR).

[45] NVIDIA TensorRT Developer Guide. https://docs.nvidia.com/deeplearning/tensorrt/developer-guide/index.html.

[46] TensorFlow Lite Guide. https://www.tensorflow.org/lite/guide.

[47] Kingma D.P., Ba J. (2014). Adam: A Method for Stochastic Optimization. arXiv.

[48] Everingham M., Van Gool L., Williams C.K.I., Winn J., Zisserman A. The PASCAL Visual Object Classes Challenge 2012 (VOC2012) Results. http://host.robots.ox.ac.uk/pascal/VOC/voc2012/.

[49] Bochkovskiy A., Wang C.Y., Liao H.Y.M. (2020). YOLOv4: Optimal Speed and Accuracy of Object Detection. arXiv.

[50] Long X., Deng K., Wang G., Zhang Y., Dang Q., Gao Y., Shen H., Ren J., Han S., Ding E. (2020). PP-YOLO: An Effective and Efficient Implementation of Object Detector. arXiv.

